# Coping strategies and associated factors among people with physical disabilities for psychological distress in Ethiopia

**DOI:** 10.1186/s12889-022-14877-0

**Published:** 2023-01-04

**Authors:** Getachew Tesfaw Desalegn, Tadele Amare Zeleke, Shegaye Shumet, Yohannes Mirkena, Tilahun Kassew, Dessie Abebaw Angaw, Endalamaw Salelew

**Affiliations:** 1 Department of Psychiatry, College of Medicine and Health Sciences, University of Gondar, P. O. Box: 196, Northwest Gondar, Ethiopia; 2 Department of Epidemiology and Biostatics Institute of Public Health, College of Medicine and Health Science, University of Gondar, Northwest Gondar, Ethiopia

**Keywords:** Coping strategy, Psychological distress, People with physical disabilities

## Abstract

**Background:**

Coping strategies are frequently used among individuals with physical disabilities when they face adversities. Low- and middle-income countries are not investigated coping styles among psychological distress persons with disabilities despite the high prevalence of psychological distress. The aim of this study was to identify coping strategies among people with physical disabilities for their psychological distress in Ethiopia has a crucial role to improve the health status of persons with physical disabilities.

**Methods:**

An institution-based cross-sectional study was employed among individuals living with physical disabilities at the University of Gondar staff and students from May to June 2021. All staff and students with physical disabilities were screened for psychological distress (*n* = 269). The census sampling technique was used to select the study participants for psychological distress. The Brief Cope with Problems Experienced (COPE-28) was used to assess coping strategies. Bivariate and multivariate linear regression analyses were used to identify factors associated with coping strategies. An odd ratio (OR) with a 95% confidence interval (CI) at *P* < 0.05 was computed to assess the strength of the association.

**Results:**

The emotional-focused coping strategy was the most frequently used when dealing with psychological distress among participants with physical disabilities. The most commonly used emotional-focused coping strategy was spirituality. In the multivariate analyses; urban residence (β = 3.05, 95% CI: 0.98, 5.12), and stigma (β = 3.10, 95% CI: 0.61, 2.83) were factors positively associated with emotion-focused coping strategy, and World Health Organization Quality of Life (WHO QOL) (β = 0.18, 95% CI: 0.13, 0.22), and stigma (β = 1.11, 95% CI: 0.61, 2.83) were factors significantly associated with problem-focused coping. Urban residence (β= -0.96, 95% CI: -1.69, -0.22) was negatively associated with dysfunctional coping strategy, but WHO QOL (β = 0.35, 95% CI: 0.32, 0.38) was positively correlated with dysfunctional coping.

**Conclusion:**

In this study revealed that spirituality is the most frequently used coping strategy among the study participants. Urban residents, stigma, and WHO QOL significantly correlated with coping strategies among such patients. The Ministry of Health, Ministry of Education, and other concerned organizations may find the present findings useful to strengthen the coping styles to minimize psychological distress among people with physical disabilities.

## Introduction

Globally, more than one billion or 15% of the world’s population are estimated to live with disabilities. About 80% of them lived in developing countries. It is more prevalent among women than men. Individuals with physical disabilities affected forty-five million people worldwide of them 90% living in developing countries [1–3]. According to the World Bank and World Health Organization(WHO) report, there are fifteen million people with disabilities in Ethiopia [4]. Throughout the world, people with disabilities have poor health outcomes, low education achievements, less financial participation, barriers in accessing services, and higher rates of poverty than people without disabilities [3, 5–7].

Disability is the umbrella term for impairments, activity limitations, and participation restrictions, referring to negative aspects of the interaction between an individual and that individual’s factors (environment and personal factors) [8]. Thus problems are a complex phenomenon, reflecting an interaction between a person’s body and the features of the society in which he/she lives [3, 9].

Coping is the ability to adjust, adapt and meet a challenge successfully. It also entails contending or dealing successfully with a challenging event [10]. Noted that coping means when one constantly changes her/his behavioral and thought effort that people adopt to master, reduce or minimize stressful events in order to manage some specific external demands that have been judged as tasking or exceeding the resources of the person [11, 12]. Or it is the reduction of tension and restoration of equilibrium [13]. There are two most commonly widely used types of coping. Coping is directed at managing or altering the problem causing the distress is problem-focused and coping is directed that regulating the emotional response to the problem is emotion-focused [13, 14]. A spiritual-focused coping strategy is finding meaning and purpose in adversity through a strong relationship with God [15].

The concept of positive coping has been associated with lower levels of psychological distress, whereas negative coping has been associated with higher levels of psychological distress [16].

It plays both independent and interactive roles in influencing physical and mental health conditions [17]. People with disabilities are confronted by using different supporting materials. These include; wheelchairs, artificial limbs, inaccessible to appropriate technology, and difficulty in repairing, and maintaining accessible devices [18]. Coping strategies are important to improve social and physical barriers to people with disabilities [19]. It is different among females and males. Males have to control stress, either overcoming or fleeing it and females are not easily cope with psychological stress due to natural conditions [20].

Different studies revealed that there are several coping strategies among individuals with disabilities for their psychological distress. These include; sought of social support, problem-solving, physical exercise, avoidance, using social media, watching movies, and relationship with others [21–23]. Social support and problem-focused coping strategies play an important role to increase life satisfaction and the personal growth of people with disabilities [24].

In Ethiopia, still unknown whether coping styles have an important impact on individuals with physical disabilities responding to psychological distress. As it is the first research attempt in Ethiopia, it is supposed to bring fresh insight into the field and serve as the basis for future researchers in the country. In Ethiopia, coping strategy has not been studied among individuals with physical disabilities living with psychological distress. Still, people who are exposed to different mental health conditions associated with their disabilities were investigated for the status of the coping styles of their mental well-being with different psychological distress, very little attention has been given to buffering psychological distress and associated factors in people exposed to different stressful events linked with their disability, which is the common problem in developing countries, and Ethiopia context particular. Therefore, the current study conducted to assess coping strategies and associated predictors among students and staff with physical disabilities at the University of Gondar in northwest Ethiopia has a vital role to overcome psychological distress by the participants and mental health professionals.

## Methods and materials

### Study design and period

An institution-based cross-sectional study design was conducted among students and staff with physical disabilities at the University of Gondar from May to June 2021.

### Study area

The University of Gondar was established in 1954, and hence this is the oldest medical training institution in the country. The University has five campuses. As we got the information from the Master card foundation and disability directorate, on all campuses around 44 masters and 178 undergraduate students with physical disabilities have been attending their classes. More than 71 individuals with physical disabilities have been employed at the University of Gondar.

### Study population

All students and staff with physical disabilities were living at the University of Gondar during the study period.

### Inclusion and exclusion

All students and staff whose age ≥ 18 years and they are living with physical disabilities were included in the study and all students who were on withdrawal and staff who were on annual/maternal/sick leave were excluded.

### Sampling technique

The census sampling technique was used to recruit the study participants at the University of Gondar. A total of 269 study samples were identified and screened for psychological distress symptoms by using a Kessler psychological distress scale (K-10). Those who scored ≥ 20 were probable psychological distress. After the screening, ninety-three participants with physical disabilities were eligible to assess their coping strategies.

### Data sources and measurements

Data were collected using an interviewer-administered structured questionnaire, which contain several other explanatory variables-including; socio-demographic factors (sex, marital status, education, occupation, residency), psychosocial factors (stigma, WHO QOL, perceived social support, suicidal behaviors, WHO Disability Assessment Schedule-2 (WHODAS-2), types of disabilities(visual, legs and others)s, clinical factors( presence of chronic illnesses), and substance use factors(Alcohol and Khat). The following instruments were employed. The coping strategy was assessed by using the Brief-COPE scale. The scale has 28 items that assess the degree to which a participant utilizes a specific coping strategy. The 28 items are being categorized into 14 coping strategies. The scale has three subscale; problem-focused, emotion-focused and avoidant coping. Respondents rate items on a 4-point Likert scale, ranging from 1 “I have not been doing this at all” to 4 “I have been doing this a lot.” It was used to assess coping styles for mental illness in our country [25–31]. In this study, cronbach’s alpha was 0.857.

We measured functional impairment using the 12 items WHODAS-2 having; six domains(cognition, self-care, getting along, life activities, mobility, and participations) that are reported the five-point Likert scale from 0 = no difficulty to 4 = very severe difficulty based on the severity of problems [32–34]. The instrument has been validated among disabilities in Ethiopian setting [35]. In this study, cronbach’s Alpha was 0.8.

Social support was assessed using the Oslo 3-item social support scale which was used in several studies. It provides a brief measure of social support and functioning and is considered to be one of the best predictors of mental health. It covered different levels of social support by measuring the number of people the respondents feel close to, the interest and concern shown by others. The Oslo-3, total scores were calculated by adding up the raw scores for each item. The score scale ranges from 3 to 14 and three broad categories: “poor social support” 3 to 8, “moderate support” 9–11, and “strong support” 12–14 [36–38].

Stigma was assessed by using an eight items of stigma scale for chronic illness (SSCI-8) [39]. It comprises eight items rated on a five-point Likert scale from one (never) to five (always). Total score range from eight to forty, with a cutoff score greater than eight indicating the presence of stigma [40, 41].

Substance related factors were assessed using WHO’s Alcohol, smoking, and substance involvement screening test (ASSSIS), and its internal consistency was in a good range (Cronbach’s Alpha = 0.80) with the sensitivity of 80%, and specificity of 71% [42].

Patients’ quality of life was assessed by using 26 items of the WHOQOL-BREF questionnaire. The questionnaire consists of two parts. The first, part evaluates the individual’s overall perceptions of quality of life and the person’s overall perception of health. The second part evaluates the four domains: physical health, psychological health, social, and environmental health. Domain scores are scaled in a positive direction (i.e. higher scores correspond to a better quality of life). The QOL raw scores are transformed into a range between 0 and 100. The overall QOL is computed as the average of the score of the four domains. The higher mean score indicates better QOL and vice versa [43].

Suicidal ideation and attempts were measured according to the WHO Composite International Diagnostic Interview (CIDI) questionnaires. If the participant provided a “Yes” answer to the question, (“During their disabilities, have you ever seriously thought about committing or attempted suicide, respectively?” they were considered to have suicidal ideation or attempt, respectively [44].

### Data processing and analysis

The completed questionnaire was checked for completeness and then was coded, recoded, and entered into Epi-info version seven statistical programs and then exported to SPSS version 21 for analyses. Both descriptive and analytical procedures were used. Descriptive statistics like frequency, percentage, mean and standard deviation (SD). After all variables fulfilled the chi-square (categorical variables), computed mean, independent sample t-test, one way ANOVA and then checked their collinearity diagnostic, and independent from other Variable Inflation factors (VIF was less than 2 and tolerance greater than 0.2 and less than 0.989) and simple linear and multiple linear regression analysis stepwise methods employed to identify factors associated with coping strategies whose *P*-values were < 0.2 level. Finally, the variables that had an independent association with coping strategies were declared based on 95% CI and *P*-value < 0.05. Model fitness was checked by using Adjusted R square from 0.43 to 0.89 at f-test 0.0001 to 0.05). An adjusted unstandardized β coefficient was used to describe the association with coping strategy.

## Results

### Socio-demographic characteristics of participants

The mean age of the respondents was 24.67 ± 5.48 years. Out of the participants, 87.1% (*n* = 81) were single, and 91.4% (*n* = 85) were Orthodox Christian followers. The majority of the study population, *n* = 81(87.1%) degree and above educational holders, nearly 90% (*n* = 81) were students and more than two-thirds of the study population got ≤ 3799 Ethiopian birrs, and more than 50% (*n* = 52) of participants were rural resident (Table 1).

**Table 1:** Socio-demographic characteristics among individuals with physical disabilities at the University of Gondar, Northwest Ethiopia, 2021(*N* = 93)

Variables	Categories	Frequency	Percent
Age	Mean (SD)	24.69(5.48)	
Sex	Male	49	52.7
	Female	44	47.3
Marital status	Single	81	87.1
	Others^a^	12	12.9
Religion	Orthodox	85	91.4
	Others^b^	8	8.6
Education	Degree and above	81	87.1
	Others^c^	12	12.9
Occupation	Student	81	87.1
	Employee	12	12.9
Income	≤ 3799	60	64.5
	> 3799	33	35.5
Residency	Rural	52	55.9
	Urban	41	44.1

^a^Others = married, separated, widowed

^b^Others = protestant and Muslim

^c^Others = primary education and secondary education

### Psychosocial and health-related characteristics of respondents

Of the participants, more than 50% (*n* = 49) had visual impairment, and one in three of the respondents had both legs disability. Nine in ten participants were stigmatized due with physical disabilities, and 55.9% (*n* = 52) had intermediate social support. A small number of *n* = 17(18.3%) and *n* = 20(21.5%) respondents were chewed khat and suicidal ideation, respectively. The mean and the standard deviation of the overall WHO QOL and WHODAS-2 were 39.1 ± 12.5 and 24.35 ± 8.25, respectively. The mean and SD of psychological distress were 26.52(5.87) (Table 2).

**Table 2:** Distribution of psychosocial and health related characteristics among individuals with physical disabilities at the University of Gondar, Northwest Ethiopia, 2021(*N* = 93)

Variables	Categories		Frequency	Percent
Types of physical disability	Visual		49	52.7
	Both legs		29	31.2
	Others^a^		15	16.1
Chronic illness	Yes		11	11.8
	No		82	88.2
Stigma	Yes		81	87.1
	No		12	12.9
Perceived social support	Poor		11	11.8
	Intermediate		52	55.9
	Good		30	32.3
Physical domain	Mean (SD)	83.52(17.3)		
Psychological domain	Mean (SD)	73.89(16.17)		
Social domain	Mean (SD)	32.6(10.33)		
Environmental domain	Mean (SD)	97.74(19.30)		
Overall WHO QOL	Mean (SD)	39.1(12.5)		
Suicidal thought	Yes		20	21.5
	No		73	78.5
Suicidal attempt	Yes		8	8.6
	No		85	91.4
Alcohol	Yes		17	18.3
	No		76	81.7
Khat	Yes		17	18.3
	No		76	81.7
Psychological distress	Mean (SD)	26.52(5.87)		
WHODAS-2	Mean (SD)	24.35(8.25)		

^a^Others = hands, and both hand and legs

### Coping strategies

The two most common coping strategies were “Giving up trying to deal with it,” and “Using alcohol or other drugs to help me get throw it.” were reported to be used ‘a lot’ by *n* = 60; 64.5% and 60.2% (*n* = 56) participants, respectively. The least frequently used coping strategies were accepting the reality of the fact and taking action from 28 item of brief COPE (Table 3). Table 4: illustrates that a brief COPE 28 item is comprised of 14 subscales, each of which assesses the degree to which a respondent utilized a specific coping strategy. Each of the fourteen scales is comprised of two items; total scores on each scale range from 2 (minimum) to 8 (maximum). Higher scores indicate increased utilization of that specific coping strategy. In this subscale, the spiritual coping style has been the most frequently used coping strategy among the respondents with physical disabilities. Table 5; illustrates the possible score of coping strategy, the sample mean coping strategy score of 41.15(SD = 11.34). The mean score of the sample can be understood as lower. The mean and SD score of problem-focused, emotion-focused, and dysfunctional coping strategies were 11.46(SD = 3.26), 13.61(SD = 5), and 15.28(SD = 4.53), respectively.

**Table 3:** Frequency of 28 items coping strategies among participants with physical disabilities for psychological distress symptoms at the University of Gondar northwest Ethiopia, 2021 (*N* = 93)

Items	Mean (SD)	Not at All	A little bit	A medium	A lot
Problem focused coping strategies	11.46(3.26)				
Thinking hard about what steps to take	2.13(0.78)	6(6.5)	5(5.4)	53(57)	29(31.2)
Trying to come up with a strategy	2.00(0.86)	1(1.1)	31(33.3)	28(30.1)	33(35.5)
Trying to get advice or help from other people about what to do	1.41(1.20)	27(29)	29(31.2)	9(9.7)	28(30.1)
Taking action to make the situation better	0.43(0.76)	64(68.8)	22(23.7)	3(3.2)	4(4.3)
Getting help from other people	2.15(1.04)	6(6.5)	26(28)	9(9.7)	52(55.9)
Concentrating my efforts on doing something about the situation I’m in	1.44(1.23)	28(30.1)	27(29)	7(7.5)	31(33.3)
Getting emotional support from others	2.00(0.89)	3(3.2)	27(29)	30(32.3)	33(35.5)
Emotion focused coping strategies	13.61(5)				
Trying to find comfort in spiritual beliefs	1.20(0.97)	28(30.1)	26(28)	31(33.3)	8(8.6)
Praying or meditating	1.65(1.07)	11(11.8)	42(45.2)	9(9.7)	31(33.3)
Looking for something good	1.12(0.91)	30(32.3)	26(28)	33(35.5)	4(4.3)
Accepting the reality of the fact	0.38(0.64)	65(69.9)	22(23.7)	5(5.4)	1(1.1)
Getting comfort and understanding	1.41(0.73)	8(8.86)	46(49.5)	32(34.4)	7(7.5)
Trying to see it in a different light to make it seem more positive	1.66(0.71)	6(6.5)	27(29)	53(57)	7(7.5)
Learning to live with it	1.77(0.96)	3(3.2)	46(49.5)	13(14)	31(33.3)
Making jokes about it	1.51(0.75)	4(4.3)	48(51.6)	31(33.3)	10(10.8)
Making fun of the situation	0.60(0.83)	54(58.1)	26(28)	9(9.7)	4(4.3)
Avoidance coping strategies	15.28(4.53)				
Watching TV, reading, daydreaming, or sleeping to think less	1.65(1.22)	29(31.2)	4(4.3)	31(33.3)	29(31.2)
Turning to work or other activities to take my mind of things	0.57(0.83)	56(60.2)	26(28)	6(6.5)	5(5.4)
Expressing my negative feelings	1.71(0.75)	5(5.4)	28(30.1)	49(52.7)	11(11.8)
Saying things to let my unpleasant feelings escape	1.75(1.07)	5(5.4)	46(49.5)	9(9.7)	33(35.5)
Saying to myself “this isn’t real	0.91(0.81)	29(31.2)	49(52.7)	9(9.7)	6(6.5)
Giving up trying to deal with it	2.32(0.97)	3(3.2)	24(25.8)	6(6.5)	60(64.5)
Giving up the attempt to cope	1.69(0.96)	4(4.3)	49(52.7)	12(12.9)	28(30.1
Refusing to believe that it has happened	1.02(0.82)	23(24.7)	52(55.9)	11(11.8)	7(7.5)
Blaming myself for things	1.53(0.73)	3(3.2)	48(51.6)	32(34.4)	10(10.8)
Criticizing myself	1.72(1.20)	24(25.8)	8(8.6)	27(29)	34(36.6)
Using alcohol to help me get through it	2.24(1.01)	5(5.4)	24(25.8)	8(8.6)	56(60.2)
Using alcohol to make myself feel better	1.15(0.91)	29(31.2)	24(25.8)	37(39.8)	3(3.2)

**Table 4:** Mean (SD) of 14 subscale coping strategies among participants with physical disabilities for psychological distress symptoms at the University of Gondar northwest Ethiopia, 2021 (*N* = 93)

Items	Mean	SD
Spiritual coping	4.56	1.89
Active coping	4	1.54
Self-distraction	3.83	1.27
Self-blaming	3.42	1.72
Planning	3.3	1.16
Emotional support	3.05	1.28
Positive reframing	3.05	1.65
Instrumental support	2.8	1.68
Acceptance	2.77	1.48
Denial	2.61	2
Venting	2.56	1.11
Behavioral	2	1.27
Humor	1.7	1.23
Substance use	0.8	1.21

**Table 5:** Descriptive statistics of coping domains among participants with physical disabilities for psychological distress symptoms at the University of Gondar northwest Ethiopia, 2021 (*N* = 93)

Coping domains	N	Mean	SD
Emotional-focused	93	13.61	5
Problem-focused	93	11.46	3.26
Dysfunctional coping	93	15.28	4.53
Coping strategy	93	41.15	11.34

### Relationship between factors and coping strategy

Sub-sample tests were formed based on the samples of categorical variables by using independent sample t-test, one-way ANOVA and post hoc pair-wise comparisons were employed to examine if a significant differences existed as the function of the variables. The independent sample t-test between female (mean = 14.68; SD = 4.66) and male (M = 12.65; SD = 5.13) produced a statistical mean difference in the emotional-coping strategy (t[90]=-1.99, *p* < 0.05), rural resident (M = 11.73; SD = 4.7) and urban resident (M = 16;SD = 4.2) yielded a statistical mean difference on emotional-coping strategy (t[89]=-4.5, *p* < 0.0001). Stigma (M = 14.22; SD = 4.72) and no stigma (M = 9.5; SD = 4.5) a statistical mean difference on emotional-coping (t[14] =-3.2) at *p* < 0.02). The independent sample t-test between stigma (M = 11.8; SD = 3.18) and no stigma (M = 9.17; SD = 2.9) produced a statistical mean difference on problem-coping strategy (t[15] =-2.7; *p* < 0.001). Finally, independent sample t-test between female (M = 16.29; SD = 4.37) and male (M = 14.37; SD = 4.5) yielded a statistical mean difference on dysfunctional coping (t[90]=-2.09; *p* < 0.04).

### Factors associated with coping strategy

In simple linear regression; sex, residence, stigma, income, suicidal attempt, alcohol use, psychological distress, WHO QOL, and WHODAS-2 were factors nominated further multiple linear regression analysis model because these predictors have satisfied preliminary assumptions to become candidate factors with coping strategy at *P* < 0.2 in simple linear regression. After controlling potential confounding factors in multiple linear regression analysis, findings showed that urban residence, stigma and WHO QOL were factors significantly associated with coping strategies *P*-value less than 0.05.

In the multiple linear regression analyses; urban residence β = 3.05(0.98–5.12), and stigma β = 3.01(1.80–7.64) were positively associated with an emotion-focused coping strategy. Stigma β = 1.11(0.61–2.83), and WHO QOL β = 0.18(0.13–0.22) were factors positively associated with a problem-focused coping strategy. Urban residence β=-0.96(-1.69-0.22) was negatively associated with dysfunctional coping strategy, but WHO QOL β = 0.35(0.32–0.38) was positively associated with dysfunctional coping strategy (Table 6).

**Table 6:** Simple and multiple linear regression of coping strategies and associated factors among respondents with physical disabilities for psychological distress at University of Gondar, northwest Ethiopia, 2021(*N* = 93)

Characteristics	Mean (SD)	Crude B(95% CI)	Adjusted B(95% CI)
Emotional focused subscale of coping			
Age		-0.13(-0.31, 0.06)	-0.06(-0.23, 0.11)
Sex			
Male	12.65(5.13)	Ref	
Female	14.68(4.66)	2.03(0.01, 4.06)	0.48(-1.55, 2.50)
Residence			
Rural	11.73(4.80)	Ref	
Urban	16(4.2)	**4.27(2.38, 6.15)**	3.05(0.98, 5.12)**
Income			
≤ 3799	13.08(4.86)	1.49(-0.64, 3.63)	0.91(-1.01, 2.88)
> 3799	14.58(5.17)	Ref	
Stigma			
Yes	14.22(4.72)	**4.72(1.80, 7.64)**	3.10(0.22, 6.00)*
No	9.5(4.98)	Ref	
Suicide attempt			
Yes	19(1.41)	-2.75(-6.39, 0.89)	-2.48(-5.80, 0.85)
No	13.49(4.98)	Ref	
Alcohol			
Yes	10.57(5.27)	3.7(1.13, 6.26)	2.07(-0.46, 4.60)
No	14.29(4.7)	Ref	
Problem-focused subscale of coping			
Sex			
Male	10.9(3.7)	Ref	
Female	12.1(2.64)	1.19(-0.31, 2.52)	0.47(-0.69, 1.61)
Residence			
Rural	10.94(3.4)	Ref	
Urban	12.12(3.0)	1.18(-0.16, 2.51)	-0.8(-2.02, 0.40)
Stigma			
Yes	11.8(3.2)	**2.63(0.70, 4.57)**	1.11(0.61, 2.83)*
No	9.17(2.89)	Ref	
Suicide attempt			
Yes	13.25(3.41)	-1.96(-4.32, 0.41)	-0.45(-2.39, 1.50)
No	11.29(3.21)	Ref	
Psychological distress		0.08(-0.03, 0.20)	-0.07(-0.09, 0.1)
WHODAS-2		0.05(-0.02, 0.13)	-0.01(-0.07, 0.07)
WHO QOL		**0.18(0.14, 2.17)**	0.18(0.13, 0.22)**
Dysfunctional coping subscale of coping			
Sex			
Male	14.37(4.52)	Ref	
Female	16.3(4.37)	1.92(0.09, 3.80)	0.14(-0.54, 0.83)
Residence			
Rural	14.19(4.23)	Ref	
Urban	16.66(4.57)	**2.47(0.64, 4.29)**	-0.96(-1.69, -0.22)*
Stigma			
Yes	15.69(4.53)	3.19(0.47, 5.91)	-0.14(-1.14, 0.86)
No	12.5(3.58)	Ref	
Psychological distress		0.11(-0.04, 0.27)	-0.01(-0.06, 0.04)
WHO QOL		**0.34(0.31, 6.37)**	0.35(0.32, 0.38)**

*=*P* < 0.05, **=*P* < 0.001

## Discussions

Coping is the expending conscious effort to solve personal and interpersonal problems and seeking to master, minimize or tolerate psychological distress associated with persons with physical disabilities. Persons with disabilities include those who have long-term physical, mental, intellectual, or sensory impairments whose interaction with various barriers may hinder their full and effective participation in society on an equal basis with others.

In this study, the overall mean coping strategy score was lower than the mean value of the total mean score of coping, but the subscale of emotional-focused coping was the highest coping strategy mean score compared with the subscale of problem-focused and avoidant coping strategies. In contrast of this study, the study did in India understanding coping with distress due to physical disabilities revealed that a problem-focused coping strategy significantly reduced the level of psychological distress [45].

Dysfunctional/avoidance coping strategies were negatively impacted psychological distress with participants with physical disabilities, which was supported by another study on physical disabilities [46]. There are positive and negative coping mechanisms used by individuals with physical challenges [47].

In this study, spiritual coping was the most frequently used coping style among study participants which was consistent with coping strategies among Poland students with physical disabilities revealing that beliefs about oneself, the world, and basic hope to contribute to explaining variations in the nature and strength of persons coping strategies [48]. In the present study, spirituality is the most frequently used coping strategy among the participants from the emotional-focused subscale. Which was supported by another study in Ethiopia, spiritual coping was the most frequently used coping strategy among psychologically distressed women [49]. Spirituality coping mechanism was significantly predictive of good mental health [50]. It has also been found to act as a resource for coping for people with physical disabilities [51]. There were many studies that supported spirituality was a good coping mechanism for psychological distress among respondents with physical disabilities [52–54]. Spirituality influences people’s ability to cope with stresses which practices are related to greater life satisfaction, happiness, positive affect, and other showing of well-being [55].

In the present study, the remarkable findings were obtained. The relationship between coping and psychological distress is not direct relation, but they might be importantly influenced by other factors [56]. Stigma was positively associated with the emotional and problem-focused coping strategies subscale. Stigma contributes to the discrimination and exclusion experienced by people with disabilities in all aspects of their lives due to lack of awareness and understanding regarding causes of disability, misconceptions about cause of disabilities often result from cultural and religious beliefs [57, 58]. Disability has its stigma pervasive in every society, but in parts of Africa and Asia, discrimination towards people with physical disabilities can be particularly oppressive. This in turn their coping styles like emotions or to solve problems were associated with disability [59]. The ability to use a positive coping strategy was connected with lower self-stigma, while negative coping strategies associated with increased stigma [60].

In the current study, quality of life among respondents with physical disabilities was positively correlated with problem-focused and avoidant/dysfunctional coping strategy subscales. Coping style can play a role in health-related quality of life associated with people with physical disabilities [46]. Quality of life was positively associated with coping style items; such as support and venting, positive reframing and acceptance, active coping, and self-distraction, in contrast, denial, humor, religion, and self-blaming were negatively associated with quality of life [61]. Coping styles correlated negatively with all quality of life domains except the mental health domain among persons with physical disabilities [62]. The quality of life and coping strategies are positively associated; supposed to be adaptive coping strategies [63] and improving the quality of life among adolescents with physical disabilities may focus on the reduction of life stress by increasing the variety of social and personal resources [64].

Another significant factor in this study, being living in the urban was correlated with emotional-focused and avoidant coping styles. Those who are living in urban were higher mean value of coping strategies than rural residents [65]. In urban adolescents have many options to solve the problem or cope with stress [65]. Rural residents was lower severity of physical distress and greater satisfaction than their counterparts from large cities [66]. Patients living with chronic medical diseases in Poland, and those who were living in rural areas had a low levels of psychological distress due to their social interaction and spirituality [66].

### Limitations

One of the most limitations of this study is social desirability bias. Despite a new qualitative study in Ethiopia, it has a small sample size of psychological distress among participants with physical disabilities. Moreover, other limitation is that it was not possible to explain the cause and effect relationship between psychological distress and coping strategies due to the cross-sectional nature of the study. The prospective study could help to elucidate whether coping styles predispose persons with physical disabilities or the consequence of psychological distress.

## Conclusion

In summary, this is the first study on coping strategies for psychological distress among respondents with physical disabilities at University of Gondar. Spiritual coping was the most frequently used coping strategy. The urban residence and stigma were positively correlated with emotional-focused coping strategies. Stigma and WHO QOL were factors significantly associated with problem-focused coping strategy and WHO QOL was positively correlated with avoidance coping style, but the urban residence was negatively associated with dysfunctional coping. The Ministry of Health, Ministry of Education, and other concerned organizations may find the present findings useful to strengthen the coping styles to minimize psychological distress among people with physical disabilities. Researchers should conduct a further study on coping styles and associated factors among persons with physical disabilities of psychological distress by using different approaches, including other study design and variables such as cohort study designs in this area as well as different parts of the county for further exploration of coping strategies.

## Data Availability

The dataset during and/or analyzed during the current study available from the corresponding author on reasonable requests.
